# An investigation into how the timing of nutritional supplements affects the recovery from post-exercise fatigue: a systematic review and meta-analysis

**DOI:** 10.3389/fnut.2025.1567438

**Published:** 2025-04-25

**Authors:** Guangxin Cheng, Zhongchen Zhang, Zhiming Shi, Yepeng Qiu

**Affiliations:** ^1^School of Sports, Southwest University, Chongqing, China; ^2^College of Physical Education and Health, Yili Normal University, Yining, China; ^3^College of Artificial Intelligence, Southwest University, Chongqing, China; ^4^National and Local Joint Engineering Research Center of Intelligent Transmission and Control Technology, Chongqing, China; ^5^College of Physical Education and Health Science, Chongqing Normal University, Chongqing, China

**Keywords:** post-exercise fatigue recovery, timing of nutritional supplementation, systematic reviews, meta-analysis, sport

## Abstract

**Background:**

This study used a systematic evaluation and meta-analysis to determine how the timing of nutritional supplements affected the recovery from post-exercise weariness. A vital component of enhancing athletic performance and advancing health is post-exercise recovery, where nutritional supplements are crucial. Although it has been demonstrated that supplementing timing may affect recovery outcomes, there is conflicting evidence about the best time to take supplements.

**Methods:**

We thoroughly searched several academic databases and screened for inclusion of randomized controlled trials, clinical trials, and observational studies that satisfied the criteria in order to examine the effects of varying nutritional supplementation timing (immediate vs. delayed supplementation) on fatigue recovery.

**Results:**

The findings demonstrated that, in comparison to delayed supplementation, protein and carbohydrate supplementation right after exercise dramatically expedited muscle recovery, glycogen recovery, and decreased tiredness. Furthermore, the impact of supplementation timing on recovery effects differed depending on the individual and the type of exercise (e.g., strength training, endurance exercise, and high-intensity interval training).

**Conclusion:**

Recovery from post-exercise weariness is significantly impacted by the timing of nutritional intake. Supplementing with protein and carbohydrates right after exercise, particularly after intense exercise, can help with tiredness relief, muscle recovery, and glycogen replenishment.

## 1 Introduction

Exercise is crucial for preserving health and enhancing physical fitness (1, 2). Nonetheless, one of the main concerns in exercise science research has been tiredness and its recovery following exercise (3, 4). In addition to reducing sports injuries and easing muscular soreness (5, 6), post-exercise fatigue recovery is a critical component influencing athletes' training efficacy and athletic performance (7, 8). Numerous physiological processes, such as muscle regeneration, energy recovery, immunological modulation, etc., are involved in the fatigue recovery process, and nutritional supplements are a useful means of hastening these processes (9–11). Protein synthesis, glycogen recovery, and hydration are the three key areas where nutritional supplements play a part in workout recovery (12–14). The timing of supplementation has drawn the attention of experts in recent years. The precise effects of immediate vs. delayed post-exercise supplementation on recovery (15–17), results have not been consistently determined (18, 19). As a result, elucidating how the timing of nutritional supplements affects recovery from post-exercise tiredness is not only valuable from an academic standpoint but also serves as a crucial guideline for athletes' recovery and sports training.

This study aims to understand how different timings of nutritional supplements affect recovery from post-exercise fatigue by meticulously examining the existing literature. It will examine the recovery effects of immediate vs. delayed supplementation under various exercise kinds, activity intensities, and participant characteristics. It will concentrate on the effects of protein, carbohydrate, and their combination supplementation. In order to offer a fresh theoretical foundation for further investigation, the physiological mechanisms that underlie the timing of nutritional supplements will also be examined.

## 2 Materials and methods

### 2.1 Literature review

#### 2.1.1 The physiological mechanisms of post-exercise fatigue

Muscular microinjuries, energy depletion, and inflammatory reactions are the primary causes of post-exercise fatigue, which is the body's physiological reaction to exercise loads and is characterized by decreased exercise capacity, muscular soreness, and physical weakness (14, 20, 21). Hydration, muscle repair, and glycogen recovery are all part of the recuperation process (22). Protein supplements are necessary to enhance muscle protein synthesis, decrease catabolism, and speed up repair of exercise-induced microdamage to muscle fibers, particularly during strength and aerobic endurance training (23, 24). Significant glycogen depletion occurs during high-intensity exercise; prompt carbohydrate administration can help to quickly replenish glycogen stores and prevent hypoglycemia and extreme exhaustion (25). Exercise causes rapid water loss due to sweating and accelerated breathing; electrolyte and water supplements help restore water balance and avoid weariness and cramping caused by dehydration (26).

#### 2.1.2 The role of nutrition in recovery

Supplementing with nutrients is crucial for the post-exercise recovery phase (27–29). The main ingredient for healing damaged muscles is protein, and taking 10–20 grams of protein supplements after working out will greatly increase muscle protein synthesis (30, 31), help lessen weariness and muscle injury (1, 32, 33). Conversely, carbohydrates aid in the recovery of glycogen, particularly during endurance and high-intensity exercise (34). According to studies, taking a carbohydrate supplement within 30 min of working out greatly speeds up the recovery of glycogen reserves and lessens fatigue (35). Short-term recovery is less affected by fat, but long-term low-intensity exercise recovery benefits from fat supplementation because it preserves bodily homeostasis (36). Supplementing with water and electrolytes is also essential to assist restore lost fluids and minerals during exercise, avoid fluid imbalance, and encourage a full recovery (37). All things considered, a key tactic to encourage quick physical recovery following exercise is the scientific and sensible supplementation of proteins, carbohydrates, fats, water, and electrolytes (38, 39).

#### 2.1.3 Theory of timing for nutritional supplementation

When it comes to when to take nutritional supplements (40), it has long been known that the 30-min period following exercise is the “golden window of recovery,” during which time muscle protein synthesis is at its peak and protein and carbohydrate supplements can hasten recovery (41, 42). According to recent research, supplementation that is delayed may occasionally have a comparable impact, particularly when it comes to low-carb diets. The best time to take supplements may vary depending on the kind, intensity, and individual characteristics of the workout (43, 44).

#### 2.1.4 Previous research and controversies

The effects of immediate vs. delayed post-exercise supplementation appear to vary, according to existing data. According to certain research, taking supplements right after working out speeds up muscle recovery, restores glycogen, and lowers inflammation (34, 45), However, some have argued that the recovery effect is unaffected by delayed supplementation (35, 46), and even lessens discomfort in the stomach. Individual variations in the timing of nutritional supplementation, variations in study design, and variations in sample characteristics are the primary causes of the disagreement surrounding this topic (47).

### 2.2 Literature selection plan and search strategy

PRISMA (Preferred Reporting Items for Systematic Reviews and Meta-Analyses) standards were applied in this work for data capture and literature scanning in order to conduct a systematic review and meta-analysis (48, 49). Web of Science, PubMed, Cochrane Library, EmBase, and MEDLINE were searched from the library's inception to March 3, 2024, to guarantee that the research material was comprehensive and of high quality. Table 1 lists the inclusion and exclusion criteria that were applied during the literature selection procedure.

**Table 1:** Inclusion and exclusion criteria used in the literature selection process.

**Inclusion criteria**	**Exclusion criteria**
Randomized Controlled Trials (RCTs) and Clinical Trials	Review articles, conference abstracts, etc.
Studies have addressed nutritional supplementation at different points in time after exercise	Studies with insufficient sample size and incomplete data
Studies evaluating post-exercise recovery effects, such as muscle function recovery, glycogen recovery, and fatigue	Non-original research, e.g., expert opinion, theoretical studies, etc.

### 2.3 Data extraction process and quality assessment

All literature was independently assessed by two researchers based on inclusion and exclusion criteria, and data extraction in this study followed highly established processes to ensure the reliability and comparability of the results (50). To prevent human bias, two separate researchers conducted the literature screening and data extraction, and the extracted data was double-checked (51). If there were any discrepancies, they were settled through discussion or by getting in touch with the original writers. The following were included in the data extraction: first, documenting the research methods and the type of study design (e.g., clinical trial, cohort study, randomized controlled trial, etc.) (52). The second step involved extracting study sample information, such as the age, gender, health status, exercise experience, kind of exercise (e.g., strength training, endurance exercise, etc.), and sample size of the participants (53). Third, interventions were meticulously documented, including the kind of nutrients provided (proteins, carbohydrates, fats, etc.), the timing of supplementation (shortly after exercise, half an hour later, etc.), and the amount (16, 35, 54). Fourth, information was gathered on important evaluation metrics, such as inflammatory response (C-reactive protein, etc.), fatigue (visual analog scale, fatigue scores), muscle recovery (muscle strength, injury markers), and glycogen recovery (blood glucose levels, muscle glycogen stores) (26, 35). Additionally, statistical techniques including *p-*values, effect sizes, and confidence intervals were noted. Standardized forms were used to extract all data, and independent reviewers double-checked the results. To guarantee the consistency and accuracy of the data, the research team would get in touch with the original writers to add to and enhance any missing information or unclear passages.

### 2.4 Statistical analysis

To thoroughly evaluate the overall impact of varying nutritional supplementation timing on post-exercise fatigue recovery, all data analyses in this study were meta-analyzed using a random-effects model. Because they can more precisely evaluate the overall effect and produce more conservative estimates, random-effects models are better suited than fixed-effects models for handling study heterogeneity (55). Because standardized mean differences (SMD) can reduce unit discrepancies among measurement instruments in various research and make cross-study comparisons easier, they were selected as the primary effect sizes. The effect size of the intervention group in comparison to the control group in terms of fatigue recovery, muscle recovery, and other factors is reflected in the SMD number for each study. The *I*^2^ statistic was employed to evaluate the heterogeneity among the studies. The degree of diversity between the findings of various research is reflected in the *I*^2^ value: a moderate to high level of heterogeneity is indicated by an *I*^2^ > 50%, while a low level is indicated by an *I*^2^ < 50%. Further sensitivity analyses will be conducted to determine whether there is a meaningful influence on the overall effect by removing studies with high heterogeneity on a case-by-case basis if the *I*^2^ value is high (>50%). Furthermore, subgroup analyses can be used to further segment the data based on essential study criteria (e.g., participant group, time of supplementation, kind of exercise, etc.) in order to investigate differences in effects among subgroups when heterogeneity is significant.

## 3 Results

### 3.1 Article selection

**Figure 1:**
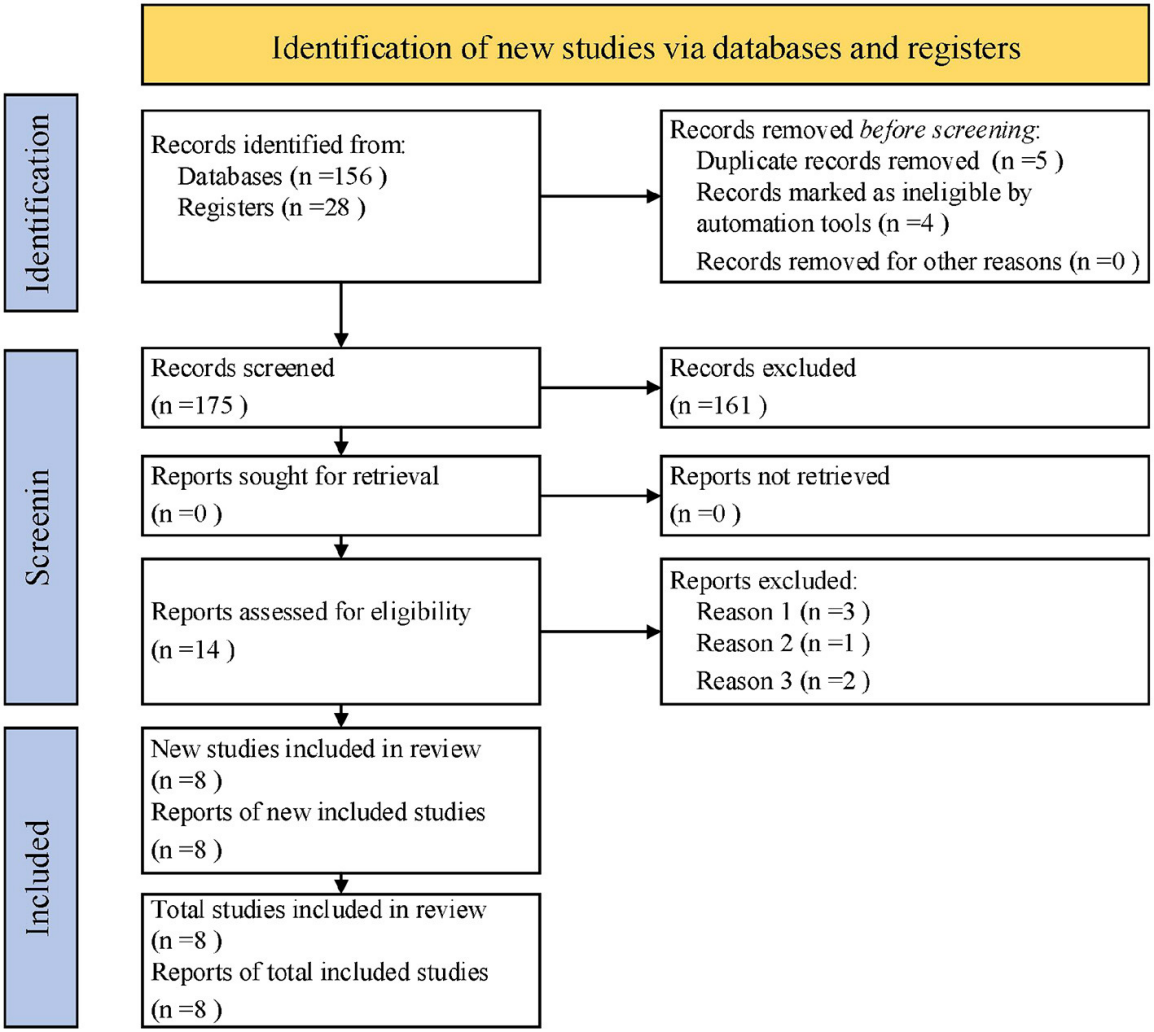
PRISMA search strategy flowchart.

PRISMA (Figure 1) standards were applied in this work for data capture and literature scanning in order to conduct a systematic review and meta-analysis (56). To guarantee the thoroughness and excellent caliber of the research literature, a multi-database search was carried out.

With a total sample size of 184, 8 (Table 2) studies that satisfied the inclusion criteria were examined. Acute exercise, aerobics, resistance training, and endurance sports were all included in the 270 patients in the sample, 158 of whom were in the experimental group and 112 in the control group.

**Table 2:** Basic characteristics of included studies.

**References**	**No. of participants**	**Type of sport**	**Timing of supplementation**	**Type of nutritional supplementation**	**Assessment indicators**	**Main findings**
White et al. (46)	27	Acute exercise	Supplementation before and after exercise	Carbohydrate-protein	Muscle damage, function and soreness	Eccentric exercise caused significant muscle damage, loss of strength, and soreness; however timing of ingestion of carbohydrate/protein supplement had no effect.
Hannaian et al. (52)	30	Aerobics	Immediate and delayed	Protein	Next-day recovery and short-term performance adaptation	Successive days of simulated team sport exercise decreases markers of next-day performance capacity with no effect of protein timing on acute recovery.
Nabuco et al. (16)	66	Resistance training	Pre-or post-exercise	Whey protein	The effect of WP pre-or post-RT on metabolic and inflammatory profile	WP pre-or post-RT promotes improvements in ALST and TC/HDL-Cratioinpre Conditioned older women. WP administered after RT was more effective in improving metabolic Health Z-score and in reducing body fat compared to placebo group.
Noh et al. (26)	8	Endurance sports	pre-exercise; half-time; mixed	carbohydrate–electrolyte	Respiratory exchange ratio, fat oxidation	CHO–electrolyte intake tests showed lower RER, RPE, and increased fat oxidation, running time, and distance than the placebo test.
Nabuco et al. (2)	70	Resistance training	Pre- or post	Whey protein	Muscle mass, muscular strength, and functional capacity	Whey protein supplementation was effective in promoting increases in SMM, muscular strength, and functional capacity in pre-conditioned older women, regardless of supplementation timing.
Snijders et al. (38)	39	Resistance exercise	Before sleep	Protein	Muscle mass and strength gains	Protein ingestion before sleep represents an effective dietary strategy to augment muscle mass and strength gains during resistance exercise training in young men.
Saunders et al. (34)	24	endurance sports	During or following	Protein	Ratings of energy/fatigue, muscle soreness and serum creatine kinase levels	Protein supplementation did not meaningfully alter recovery during the initial 24 h following a marathon. However, ratings of energy/fatigue and muscle soreness were improved over 72 h when CP was consumed during exercise, or immediately following the marathon.
Tipton et al. (17)	6	Resistance exercise	Pre- or post	Acid-carbohydrate	Muscle protein synthesis; muscle protein breakdown	The response of net muscle protein synthesis to consumption of an EAC solution immediately before resistance exercise is greater than that when the solution is consumed after exercise, primarily because of an increase in muscle protein synthesis as a result of increased delivery of amino acids to the leg.

WP, whey protein; RT, resistance training; CHO–electrolyte, carbohydrate–electrolyte; RER, respiratory exchange ratio; RPE, ratings of perceived exertion; SMM, skeletal muscle mass; CP, carbohydrate protein; EAC, essential amino acid-carbohydrate supplement.

Three US studies, two Brazilian studies, one Korean study, one Canadian study, and one Dutch study were included in the literature, which was published between 2001 and 2023. Serum creatine kinase levels, muscle protein synthesis and catabolism, muscular pain and recovery, and the impact of whey protein on inflammation and metabolism were among the evaluation criteria. There is less uniformity in the selection of assessment indicators due to the diversity of experimental selection and post-exercise recovery evaluation indicators, which could skew the assessment outcomes.

The research design in supplementation strategy and timing varies greatly, and the time nodes of post-exercise supplementation are inconsistent. A portion of the studies divide supplementation timing into two groups: immediate supplementation and delayed supplementation. Another portion further subdivides supplementation into supplementation at 30 min, 1 h, and 2 h post-exercise, which may also affect the assessment results.

### 3.2 Meta-analysis and meta-regression results

**Figure 2:**
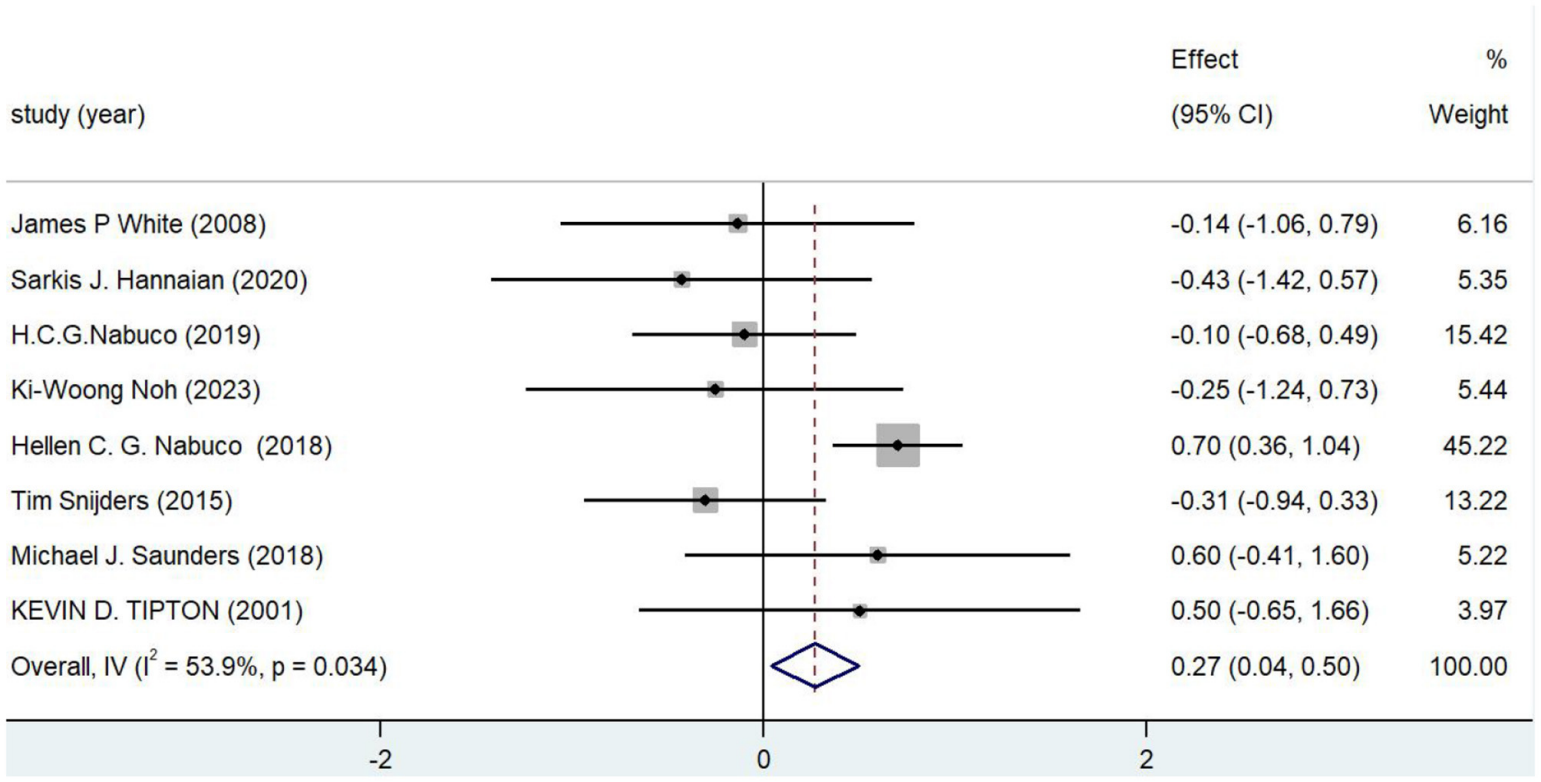
Forest map.

The timing of nutritional supplements had some positive effects on the major indices of post-exercise fatigue recovery and attained statistical significance, according to the results of the meta-analysis (Table 3) and the forest plot (Figure 2). With a *p-*value of 0.022 and a 95% CI of [0.039, 0.498], the combined effect value (Overall IV) was 0.269. Overall, the effect was significant (*p* < 0.05), suggesting that nutritional supplementation improved post-exercise fatigue recovery in a statistically significant way.

**Table 3:** Meta-analysis of different supplementation timing on recovery from post-exercise fatigue.

**References**	**Effect**	**[95% Conf. interval]**		**% Weight**
White et al. (46)	−0.136	−1.061	0.789	6.16
Hannaian et al. (52)	−0.427	−1.420	0.565	5.35
Nabuco et al. (16)	−0.099	−0.684	0.486	15.42
Noh et al. (26)	−0.253	−1.237	0.732	5.44
Nabuco et al. (2)	0.703	0.362	1.045	45.22
Snijders et al. (38)	−0.305	−0.937	0.326	13.22
Saunders et al. (34)	0.598	−0.406	1.603	5.22
Tipton et al. (17)	0.504	−0.649	1.657	3.97
Overall, IV	0.269	0.039	0.498	100.00

A *p-*value of 0.034 and an *I*^2^ of 53.9% indicated a considerable level of study heterogeneity. Heterogeneity may result from variations in the interventions (dosage, time point, kind of supplementation), sample characteristics (gender, age, activity style, etc.), or methods of assessment used in different research.

With the highest study weight (45.22%) and a positive impact value (0.70, 95% CI [0.36, 1.04]), the study by Nabuco et al. (2) appeared to demonstrate that supplementation was useful in enhancing fatigue recovery. Although the effect sizes of the studies by Saunders et al. (34) and Tipton et al. (17) were reasonably large, their CI ranges were wide, and their findings were more unclear. The fact that several of the studies [such Noh et al. (26) and White et al. (46)] had effect values near zero and CI spanning zero indicates that they did not demonstrate a substantial effect.

### 3.3 Subgroup analysis

**Figure 3:**
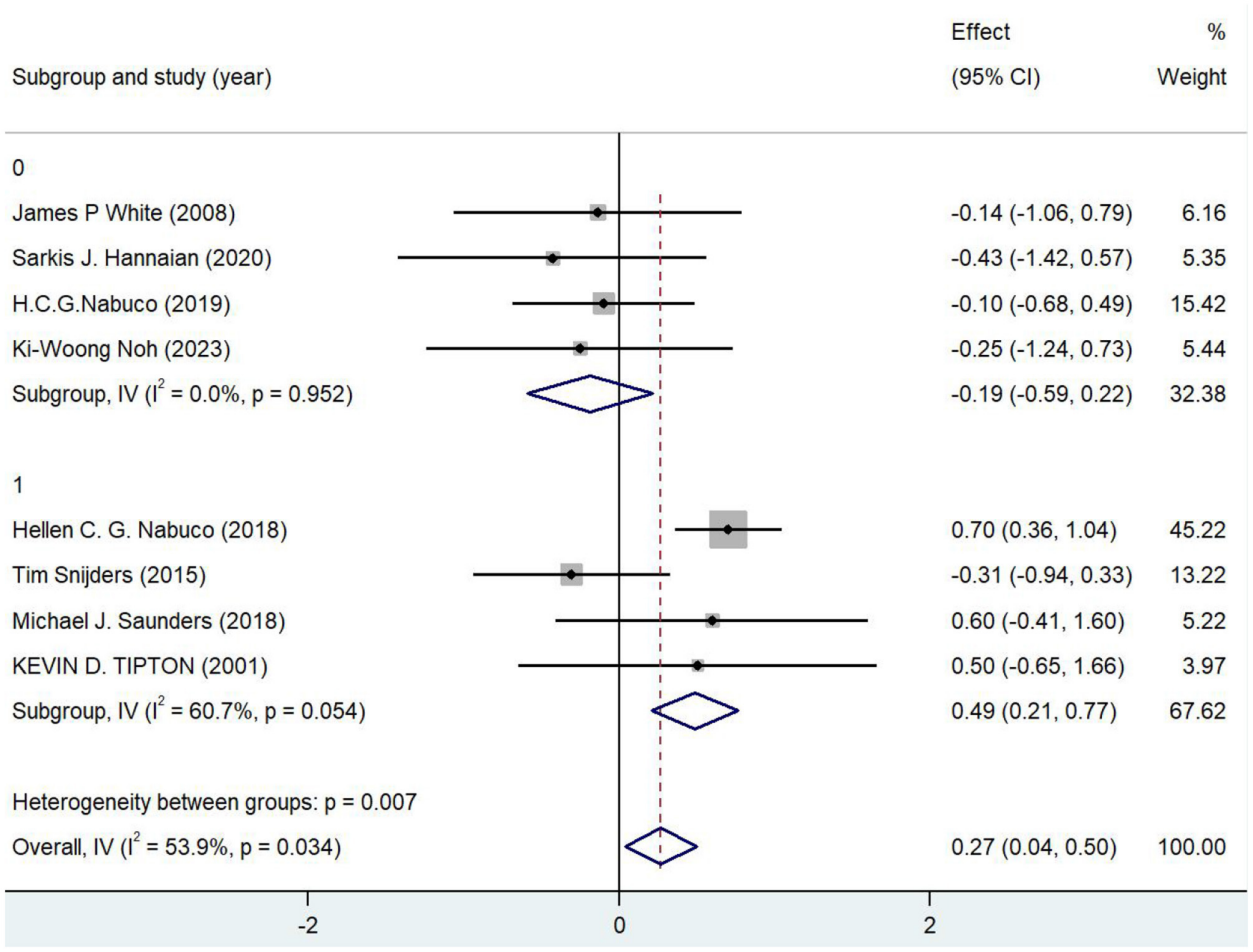
Forest map for subgroup analysis.

Subgroup studies showed (Figure 3) that the timing of nutritional supplementation had a substantial impact on the recovery from post-exercise tiredness. Although there was some heterogeneity (*I*^2^ = 53.9%), the overall analysis demonstrated that nutritional supplementation significantly improved fatigue recovery (combined effect value of 0.269, 95% CI [0.039, 0.498], *p* = 0.022). With no significant effect and no heterogeneity (*I*^2^ = 0%), subgroup 0′s combined effect value was −0.186 (95% CI [−0.590, 0.218]), *p* = 0.366. This suggests that this subgroup might reflect some of the less successful supplementation techniques or less ideal supplementation timing. With a high degree of internal heterogeneity (*I*^2^ = 60.7%) and a combined effect value of 0.486 (95% CI [0.207, 0.766]), *p* = 0.001, subgroup 1 demonstrated a significant positive effect. This suggests that subgroup 1 consists of more effective supplementation strategies, such as timely protein supplementation or particular nutrient combinations after exercise. The significant differences in effects between subgroups are further confirmed by the *p-*value of 0.007 for the test of heterogeneity between subgroups, highlighting the crucial role that supplements type and timing play in fatigue recovery efficacy. While subgroup 0 may have a weaker effect because of things like later or earlier supplementation timing and ineffective ingredients, subgroup 1′s positive results contributed more to the overall effect (weight of 67.62%), demonstrating the efficacy of timely post-exercise supplementation. In order to improve the results' applicability and generalizability, future research should further optimize supplementation timing points and ingredient selection, as well as thoroughly examine participant characteristics and other potential factors that may influence the effect. Overall, the subgroup analyses emphasized the significance of supplementation timing and strategy.

## 4 Discussion

### 4.1 Main findings

According to the study's findings, the timing of nutritional supplements is essential for the recovery from post-exercise weariness (33). Following exercise, immediate supplementation is beneficial for reducing fatigue and enhancing muscular function, particularly when paired with protein and carbohydrates (57). Conversely, delayed supplementation had comparable results to immediate supplementation, particularly in specific groups (such as those following low-carb diets) (52).

The results of this study further support the effect of timing nutritional supplementation on recovery, especially following high-intensity exercise, when compared to previous research. In contrast, Jozo Grgic found that post-exercise supplementation with sodium bicarbonate significantly improved muscular endurance but had no significant energizing effect on muscular strength (53), and Danielle T found no difference between male and female genders when supplementing with branched-chain amino acids (BCAAs) up to 24 h post-exercise (20). These findings demonstrate that delayed supplementation is not less effective than immediate supplementation under certain specific conditions. Individual variances, supplement dosage, activity style, and supplement type may all have a significant impact on this.

### 4.2 Mechanism exploration

On the one hand, by affecting biology, the impact of replenishment timing on recovery may be realized. The effects of immediate and delayed post-exercise supplementation on muscle repair processes, metabolic control, and neuroprotection can vary. Immediate AKG (α-ketoglutarate) supplementation during high-intensity training facilitates quick entry into the tricarboxylic acid cycle (TCA cycle), aids in amino acid metabolism, and blocks the mTOR pathway, which lowers fatigue and stabilizes blood ammonia levels (58). Neurotrophic factor supplementation that is delayed can decrease pain and inflammatory responses and modify damage receptor sensitivity (e.g., NGF and GDNF) (59).

On the other hand, by affecting physiological processes, the timing of supplementation may have an impact on recovery. Muscle protein synthesis is stimulated and muscle damage is decreased by taking protein supplements right after exercise. Supplementing with carbohydrates on time aids in the quick restoration of glycogen stores and lessens tiredness. Supplementing right after exercise may hasten healing by lowering inflammatory reactions (60).

### 4.3 Research limitations and future research directions

Some studies did not strictly adhere to the randomized controlled trial design, which could introduce bias; some studies had insufficient data, which impacted the accuracy of the meta-analysis; The results of the combined meta-analysis may have been biased due to the inadequate inclusion of literature on different forms of nutritional supplementation for post-exercise recovery, particularly on the timing of post-exercise nutritional supplementation and sample characteristics; exercise types; Only muscle soreness and recovery, serum creatine kinase levels, muscle protein synthesis and catabolism, and the impact of whey protein on metabolism and inflammation were utilized in this paper due to the large number of post-exercise recovery assessment indicators; these will be examined in greater detail in subsequent research; and supplementation strategies varied among the included studies, which could impact the consistency of the results. Future research could look into how individual characteristics (e.g., training level, dietary habits) and different exercise types and intensities influence when to take supplements. To assess the long-term effects and processes of supplement scheduling, more thorough, long-term clinical trials are required.

## 5 Conclusions

The timing of dietary intake has a major impact on recovery from post-exercise fatigue. Taking protein and carbohydrate supplements immediately following physical activity, especially vigorous exercise, can aid in muscle healing, glycogen replacement, and fatigue alleviation. For athletes and the broader exercise population, the study described in this paper provides evidence-based recuperation strategies. Future studies should concentrate on figuring out when different persons and activity scenarios benefit from taking supplements.
